# Prostate Biopsy: What is the future of this old Procedure? The Multiparametric Magnetic Resonance Imaging MRI/TRUS fusion prostate biopsy

**DOI:** 10.1590/S1677-5538.IBJU.2015.05.01

**Published:** 2015

**Affiliations:** Professor Associado da Unidade Urogenital, Universidade Estatual do Rio de Janeiro, Urologista de Lagoa Hospital Federal

The September-October 2015 issue of the International Braz J Urol presents original contributions with a lot of interesting papers in different fields: Infertility, Kidney Stones, Pediatric Urology, Uro-Gynecology, BPH, Prostate Cancer, Renal Cancer, Sexual Dysfunction and basic research. The papers come from many different countries such as Brazil, USA, Italy, Korea, Thailand, Turkey, Iran, Mexico, Spain and India as usual the editor´s comment highlights some papers. We decided to comment about prostate biopsy, because we had 4 papers about this topic in this issue.

Doctor Pepe and collegues, from Italy performed on page 844 an interesting study about the anterior prostate biopsy. They studied 400 patients with negative digital rectal examination and PSA values > 10 ng/mL and PSA between 4.1–10 or 2.6–4 ng/mL with free/total PSA ≤ 25% and ≤ 20%, respectively. They concluded that Anterior zone (AZ) biopsies increased detection rate for prostate cancer (PCa) (10% of the cases), the majority of AZ PCa with histological findings predictive of clinically significant cancer were found at repeat biopsy (about 70% of the cases).

Doctor Temiz and collegues, from Turkey performed on page 859 an elegant study about local anesthesia and the detection rates of prostate cancer in Transrectal prostate biopsy. The authors studied 422 patients underwent 10 core-TRUS-Bx because of elevated serum prostate specific antigen (PSA) level (>2.5ng/mL) and/or suspicious digital rectal examination findings. Patients were divided into two groups according to the applied anesthesia technique: intrarectal lidocaine gel anesthesia (IRLA) and periprostatic nerve blockade (PNB) techniques. They concluded that PNB is superior to IRLA in terms of cancer detection rates.

Doctor Ucer and collegues, from Turkey performed on page 864 a study about PSA-Age volume score in predicting positive prostate biopsy findings. The authors performed 4717 prostate biopsies. PSA-age volume was calculated by multiplying the patient's age by the prostate volume and dividing it by the PSA level. Sensitivities and specificities of the PSA-AV were assessed by retrospective analysis of findings from 4,717 prostate biopsies. The authors concluded that the sensitivity and specificity of a PSA-AV of 700 for predicting positive biopsy findings were similar to a PSA of 4ng/mL. They suggested the PSA-AV cut-off of 700 should only be used in patients younger than 60 with low prostate volumes (<20cm3).

Doctor Bulut and collegues, from Turkey performed on page 906 an interesting study about the efficacy of duration of prophylactic antibiotics in transrectal ultrasound guided prostate biopsy. The authors studied 367 patients undergoing a prostate biopsy and divided into 2 groups according to prophilaxy: oral ciprofloxacin (750 mg every 12 hours) for 3 or more days in Group-1 and single day in Group-2 and concluded that in a selected patient population single dose prophylaxis with ciprofloxacin can be safely administered compared to other regimens of 3 or more days. Increasing the duration of antibiotic prophylaxis does not decrease infectious complications.

The 4 papers are very interesting but what is the future of the prostate biopsy? The answer is Multiparametric Magnetic Resonance Imaging MRI/TRUS fusion prostate biopsy.

Multiparametric Magnetic Resonance Imaging (MRI) is emerging as a powerful test to diagnose and stage prostate cancer (PCa) (1). Prostate Imaging Reporting and Data System version 2 (PI-RADSv2) correctly identified 95 % of PCa foci ≥ 0.5 mL, but the PI-RADSv2 was limited for the assessment of GS ≥ 4+3 tumours ≤ 0.5 mL (2). Quantitative MRI parameters can predict malignant histology on MRI/TRUS fusion prostate biopsy, which is a valuable technique to ensure adequate sampling of MRI-visible suspicious lesions under TRUS guidance and may impact patient management (3).
